# Cataract Surgical Outcomes In Diabetic Patients: Case Control Study

**DOI:** 10.4103/0974-9233.53868

**Published:** 2009

**Authors:** Oluwatoyin H. Onakpoya, Charles O. Bekibele, Stella A. Adegbehingbe

**Affiliations:** Ophthalmology Unit, Department of Surgery, Obafemi Awolowo University, Ile Ife, Nigeria; 1 Department of Ophthalmology, University College Hospital, Ibadan, Nigeria

**Keywords:** Cataract, cataract extraction, diabetes mellitus, diabetic retinopathy, visual outcome

## Abstract

**Purpose:**

To determine the visual outcome of cataract surgery in diabetes mellitus with advanced cataract in a tertiary institution in Nigeria.

**Design:**

A retrospective case control study conducted at the University College Hospital, Ibadan Nigeria.

**Subjects:**

Twenty three consecutive patients with diabetes and 23 age and sex matched non-diabetic control patients who had extracapsular cataract extraction for advanced cataract between 2002-2005.

**Main outcome:**

Mean post operative visual acuity and surgical complications.

**Results:**

Twenty three patients with diabetes mellitus and 23 non diabetic controls were studied; mean duration of diabetes was 8.1 ± 7.2 years. The mean post operative visual acuity in diabetics was 0.11±0.38, 0.33±0.57 and 0.38±0.49 at one week, two months and six months compared with 0.23±0.19, 0.46±0.37 and 0.48±0.31 in non diabetics. (p=0.207, 0.403 and 0.465 respectively). Improvement in preoperative visual acuity was noted in 84.2% and 90% in diabetics and non-diabetics respectively. Poor visual outcome in diabetics was mainly due to diabetic retinopathy, maculopathy or diabetes related surgical complications.

**Conclusion:**

Visual improvement was seen following surgery for advanced cataract in diabetics in this study population. Post operative monitoring for treatment of diabetic retinopathy may enhance visual outcome.

## INTRODUCTION

Diabetes mellitus is a risk factor for development of cataract.^1^ Development of cataract is the second most ocular common complication of diabetic mellitus.^2^ Up to 20% of all cataract surgery in the United Kingdom is performed in diabetics.^3^ Furthermore epidemiologic data suggests that there is an increasing incidence of diabetes mellitus in developing countries.^4^ Cataract surgery in diabetics is indicated for visual improvement or to allow assessment and treatment of retinopathy.^5^ Poorer visual outcome in diabetics has been linked with the severity of retinopathy and maculopathy prior to cataract surgery.^1^,^2^,^6^ Cataract surgeries are often carried out earlier amongst diabetics in developed countries to allow diagnosis and treatment of retinopathy and maculopathy.^7^

In contrast, in developing countries, mature and hypermature cataracts presenting for surgical care is the rule rather than the exception.^8^,^9^ A large proportion of patients would not have had an ophthalmic examination until they present in the eye clinic with advanced cataract for surgery;^9^ thus preventing preoperative retinal assessment or treatment of retinopathy. The increasing incidence of diabetes in developing countries such as Nigeria necessitates an assessment of the surgical outcome of diabetic cataract among the study population. This study was carried to investigate the outcome of surgery in this cohort and also with the intention of making recommendations for improved care.

## MATERIALS AND METHODS

The study was carried out at the University College Hospital, Ibadan a tertiary health care institution in southwest Nigeria. This retrospective study was conducted in accordance with the ethical standards of Institutional Ethical Review Committee.

The cases consisted of consecutive diabetic patients who underwent cataract surgical procedure between January 2002 and December 2005 (Group I). All surgeries were performed by consultant ophthalmologists. The controls were age and sex matched non-diabetic patients who had cataract extraction during the same period (Group II) by consultant ophthalmologists. The diagnosis of diabetes was based on fasting blood sugar of >120mg/dl. Patients with traumatic, uveitic or complicated cataracts were excluded. All patients routinely had preoperative fasting blood glucose analysis within one week of the surgery. Glycemic control level in Group I patients was regarded as good (<70mg/dl), moderate (70-100mg/dl) or poor (>100mg/dl). Extracapsular cataract extraction with posterior chamber intraocular lens implantation (ECCE/PCIOL) under peribulbar anaesthesia was the procedure of choice. Group I patients were co-managed with endocrinologists. None of the patients had biometry for IOL power calculation as facilities for such were not available.

The duration of diabetes, method of treatment, systemic and ocular co-morbidities were recorded. Preoperative best visual acuity, intra-operative and post-operative complications as well as presenting visual acuity at 1 week, 4 weeks, 2 months and 6 months post cataract extraction was noted for all patients. The Snellen acuity was recorded as decimal number and presented as mean and standard deviation of the visual acuity during postoperative period.

Data was imputed and analyzed using the SPSS version 11 (Chicago, IL). Statistical significance was inferred at p<0.05 and p values were determined using chi square or ANOVA tests as appropriate.

## RESULTS

Forty-six patients, 23 diabetic and 23 age and sex matched non diabetic controls constituted the study population. The age and sex distribution patients were similar with a mean age of 59.3±16.8 years and 58.7±17.4 years in diabetics and non-diabetics respectively (p=0.92). The preoperative best visual acuity were <3/60 in all patients (100%) in diabetics and in 22 (95.7%) patients in non-diabetics. A mature cataract was present in all patients of both groups precluding examination of the posterior segment. (Table 1)

**Table 1:** Characteristics of the study population

	Diabetic	Non diabetic
Number of patients	23	23
Male/Female (m:f)	19/4 *(4.8:1)*	20/4 *(5:1)*
Mean age (yrs)*	59.3±16.8	58.7±17.4
Preoperative BVA < CF#	23	22
Type of cataract	mature (23)	mature (22)
		hypermature (1)

* *P= 0.92*,

# Counting fingers

The duration between the diagnosis of diabetes and cataract surgery ranged from 6 months to 20 years with mean of 8.1 ± 7.2 years. Most (82.6%) patients had Type II diabetes while the remaining 4 (17.4%) were Type I diabetics. Oral hypoglycemic agent was the method of treatment in 16 (69.9%) patients, insulin in 6 (26.1%) while 1 (4.3%) patient was on dietary control only. The immediate preoperative glycemic control was good in 6 (26.1%), moderate in 14 (60.9%) and poor in 3 (13.0%) of group 1 patients.

The ocular co-morbid diseases were similar in both groups. Hypertension was the most frequent systemic co-morbid disorder in both groups; it was more frequent in diabetics (60.9%) compared with 26.1% in non diabetics (p=0.017). (Table 2)

**Table 2:** Frequency of co-morbid systemic and ocular disease in patients

Disease	Frequency (numbers)	
	
	Diabetics	Non-diabetics
Systemic		
Hypertension	14	6
Renal impairment	2	-
Peptic ulcer disease	1	-
Chronic obstructive airway disease	1	-
Ocular		
Age related macular degeneration	1	1
Primary open angle glaucoma	1	1
Pterygium	1	-
Total	21	8

Good visual outcome was recorded more commonly in group 2 patients when compared with group 1. However, these differences were not statistically significant throughout the postoperative period (Table 3). The mean post operative visual acuity at 2 months postoperative period was 0.33±0.57 and 0.46±0.37 for groups 1 and 2 respectively (*P=0.40*).

**Table 3:** Post operative visual acuity

Post operative period	Best visual acuity		*P value
		
	Diabetics	Non-diabetics	
1 week	0.11 ± 0.38	0.23 ± 0.19	0.207
4 weeks	0.26 ± 0.59	0.35 ± 0.25	0.500
2 months	0.33 ± 0.57	0.46 ± 0.37	0.403
6 months	0.38 ± 0.49	0.48 ± 0.31	0.465

* ANOVA

The post operative visual acuity improved in 16 (84.2%), remained the same in 2 (10.5%) and was worse in 1(5.3%) patient in Group 1 whereas improvement was seen in 18 (90%) and remained the same 2(10%) patients in group 2. The visual outcome was poor (<6/60) at 2months postoperatively in 6 (30%) of 20 patients in Group I and in 2 (10.5%) of 19 patients in group 2. The reason for poor outcome was noted with diabetic retinopathy or maculopathy accounting for 33% in group 1 (Table 4).

**Table 4:** Reasons for poor BVA (>6/60) at 2 months post cataract extraction

Reasons	Frequency	
	
	Diabetics	Non-diabetics
Diabetic retinopathy/ maculopathy	2	-
Age related macular degeneration	1	1
Endophthalmitis		1
Optic atrophy	-	1
Fibrinous exudates	1	-
Retinal detachment	-	-
Posterior capsule opacity	1	-
Total	6	2

Intra-operative complications were more frequent in Group 1 patients (p = 0.001) with posterior capsular rent being the most frequent complication. Post operative complications were also more frequently noted among Group 1 patients (Table 5).

**Table 5:** Frequency of Surgical Complications

Complications	Frequency	
	
	Group I	Group II
Intra-operative complications		
Posterior capsular rent	6	2
Vitreous loss	3	-
Hyphema	1	1
Post-operative complications		
Pigment dispersion	6	1
Striate keratopathy	7	3
Fibrinous exudates	4	1
Raised intraocular pressure	2	-
Posterior capsular opacity	2	1
Wound dehiscence	1	-
Intraocular lens displacement	-	1
Endophthalmitis	1	-
Total	33	10

## DISCUSSION

In this study, the indication for surgery in all patients was for visual improvement or when the density of lens opacity was severe enough to prevent retinal assessment. Advanced cataract presenting for surgical removal is still widespread in this region similar to that reported previously.^9^ This poses a challenge in management of this group of patients since diabetic maculopathy and retinopathy are reasons for poor visual outcome following cataract surgery in diabetics.^1^,^2^,^6^ In fact, cataract surgery ought to be performed earlier amongst diabetics compared to their non diabetic counterparts to ensure timely assessment of the retina.^5^

The mean duration of diabetes mellitus prior to surgery of 8.1 years in this study is lower than 13 years reported by Squirell *et al*.^10^ Patients present late for medical care in developing countries^11^ with subsequent delay in disease diagnosis. Thus, the reported mean age at presentation and duration of diabetes prior to surgery may be a reflection of delay in diagnosis rather than the duration of the disease. Immediate preoperative glycemic control was poor in 13% of our patients. Nascimento *et al.* reported that serum glucose level had no influence on the peri-operative clinical complications and final visual outcome of cataract surgery amongst diabetic patients.^12^ Rapid pre-operative glycemic control should be avoided as it may increase the risk of postoperative progression of retinopathy and maculopathy.^13^

Systemic hypertension though the most frequent systemic co-morbid disease in both groups was more frequent amongst diabetics as seen in 60.9% compared with 26.1% of non diabetic counterparts (p=0.017). A similar high incidence of hypertension of 58% amongst diabetics for cataract surgery has been reported.^10^

The post-operative visual acuity was worse throughout the post-operative period amongst diabetics. This differs slightly from a previous report in which visual acuity was worse only by 7^th^ day and 6^th^ months post cataract extraction.^2^ In this study the difference in visual outcome among the two groups was however not statistically significant through the study period. Improvement in the post operative visual acuity was noted in 84.2% and 90% of diabetics and non-diabetics respectively. This finding supports previous reports that diabetic patients, those with maculopathy and retinopathy may have valuable visual improvement after cataract surgery.^5^

Extracapsular cataract extraction with intraocular lens implantation is well tolerated in diabetics^7^ with an overall good visual outcome^1^ as evidenced by an 84.2% rate of improvement in preoperative visual acuity. A slightly worse visual outcome was recorded amongst diabetics as evidenced by the higher percentage of patients with poor outcome. The poor visual outcome was largely due to diabetic retinopathy or maculopathy in 33% and increased incidence of post operative complications in another 33%. This differed from the non diabetic patients in whom poor visual outcome was due to preexisting ocular morbidities of AMD, optic atrophy and retinal detachment. Preoperative identification and laser treatment of diabetic retinopathy are known to improve final visual outcome in such patients.^1^,^2^,^5^,^6^ A previous study suggests that only one-third of eyes with diabetic retinopathy presenting with advanced cataract could be identified with pre-operative ultrasonography.^9^ Hence this is not a recommended solution for routine preoperative posterior segment assessment in this group of patients. We suggest that diabetic patients presenting with advanced cataract for extraction will benefit from prompt post operative assessment and treatment of retinopathy.

Complications were more frequent among diabetic patient's especially posterior capsular rent, striate keratopathy and fibrinous exudation. Menchini *et al.* reported intraocular inflammation and its sequelae as the most common complication in their study.^1^ Ivancic *et al.* reported that inflammatory reactions and bleeding which resulted in post-operative keratopathy, fibrinous uveitis and posterior capsule opacity as the common complications of cataract surgery amongst diabetics.^2^ A case of acute endophthalmitis was noted in a diabetic patient who missed 1 week post operative follow up and presented 3 weeks postoperatively with a large wound dehiscence and acute bacterial endophthalmitis. The visual acuity of hand movement in this patient throughout the study period would have contributed to a worse visual outcome we observed among diabetic patients.

Diabetics with advanced cataract presenting for cataract surgery have an overall good visual outcome and should not be denied surgery. However, extra precaution needs to be taken intra-operatively as well as adequate post operative monitoring is recommended. Also treatment of existing diabetic retinopathy or maculopathy should be performed to improve visual outcome.
